# Fast quantification of fluoroquinolones in environmental water samples using molecularly imprinted polymers coupled with internal extractive electrospray ionization mass spectrometry

**DOI:** 10.1039/c8ra01837e

**Published:** 2018-05-11

**Authors:** Wei Kou, Hua Zhang, Aisha Bibi, Mufang Ke, Jing Han, Jianliang Xiong, Rui Su, Dapeng Liang

**Affiliations:** State Key Laboratory of Inorganic Synthesis and Preparative Chemistry, College of Chemistry, Jilin University Changchun 130012 China; Changchun University of Chinese Medicine Changchun 130117 China sranalchem@163.com; State Key Lab of Groundwater Resources and Environment Ministry of Education, Jilin University Changchun 130012 China liangdp@jlu.edu.cn; School of Pharmaceutical Sciences, Peking University Beijing 100191 China

## Abstract

In this study, a facile method based on molecularly imprinted polymers (MIPs) combined with internal extractive electrospray ionization tandem mass spectrometry (iEESI-MS/MS) was developed for the quantitative analysis of fluoroquinolones (FQs) in environmental water samples. FQ molecules in water samples were captured by the MIPs, which was retained on a 0.22 μm syringe filter. Then, an electrospray solution selected as the elution solution was employed to extract the FQs from the MIPs, getting an eluate of FQs for mass spectrometric interrogation. Under the optimized experimental conditions, low limits of detection (LODs, 0.015–0.026 μg L^−1^), with relative standard deviations (RSDs) less than 8.81% (*n* = 6) were obtained. The present method also provides good recoveries (91.14–103.60%) with acceptable precision (RSDs < 6.18%) and have no serious matrix effects for environmental water samples. The experimental results demonstrated that MIPs-iEESI-MS/MS has advantages including easy use, high speed (less than 3 min per sample) and high sensitivity for the analysis of FQs in environmental water samples, showing potential application in environmental science and water safety control.

## Abstract 2

A simple, fast and high-sensitivity method for quantification of fluoroquinolones in environmental water samples using MIPs-iEESI-MS.

## Graphical abstract

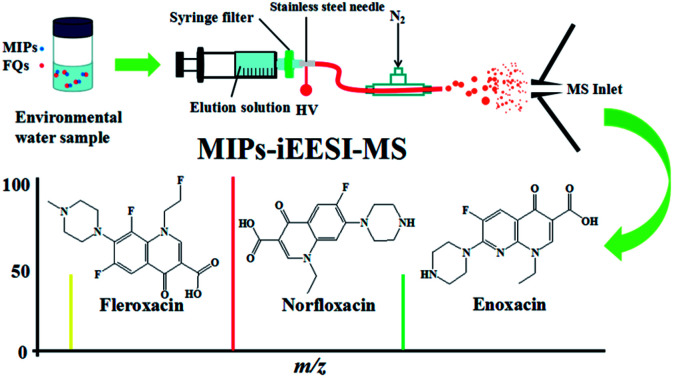

## 1. Introduction

Fluoroquinolones (FQs) have been widely used in the treatment of bacterial infections.^1–4^ However, some of the FQs cannot be absorbed completely, and are excreted through urine and excrement.^5^ Thus, FQs have been found in soil, environmental water and even in drinking water.^6^ FQs contaminations create a serious threat to human health due to their harmful effects, such as drug resistant pathogens and causing allergic reactions.^7–12^

Numerous methods have been developed for the determination of FQs in environmental water, bio-fluids, and foodstuffs *etc.*, including electrochemical methods,^13^ liquid chromatography tandem mass spectrometry (LC-MS/MS),^14,15^ high performance liquid chromatography with ultraviolet detection (HPLC-UV),^16,17^ high-performance liquid chromatography coupled to photodiode array (HPLC-PDA),^18^ and enzyme immunoassays.^19,20^ However, laborious sample pretreatments (*e.g.*, centrifugation and chemical pre-extraction, *etc.*) and large volumes of organic solvents are usually required in the analysis of complex environmental water samples. Therefore, a highly sensitive, efficient, and simple method is needed. In recent years, molecularly imprinted polymers (MIPs) materials have been employed in classical solid-phase extraction and solid-phase microextraction,^21,22^ due to their unique structure with ideal recognition sites.^23–25^ MIPs are synthesized by the cross-linking of functional monomers and specific template molecules. Three-dimensional cavities, which can specifically capture the template molecule, are left on the polymers after removal of the templates, which makes MIPs high selective for the capture of specific compounds in complex systems.^26^

Recently, ambient mass spectrometry (AMS) such as desorption electrospray ionization (DESI),^27^ extractive electrospray ionization (EESI),^28^ microwave plasma torch (MPT),^29^ direct analysis in real time (DART)^30^ and paper spray ionization (PSI),^31^ which allows the direct analysis of raw sample in atmospheric environment with high sensitivity and requires few or no sample pretreatment, has received wide attention. Internal extractive electrospray ionization (iEESI) was developed to analyse the interior of the bulk sample.^32^ In iEESI, a charged extraction solution is directly infused through the whole-volume of a bulk sample. The analytes were extracted and move toward the sample edge that faces the ion entrance of the mass spectrometer with the solvent flow. A charged plume is formed at the edge of the sample. With the help of nitrogen gas, gas phase ions are analyzed by MS. Intrinsically, iEESI has the advantages of high sensitivity, good tolerance of matrices, simple maintenance, easy operation and low cost. Up until now, iEESI-MS has mainly been applied for the qualitative characterization of various samples, such as tissue and fruit.^33,34^

In this paper, a novel and facile method, termed MIPs extraction coupled with online iEESI-MS/MS elution and analysis (MIPs-iEESI-MS/MS) was designed and developed for the fast quantification of FQs in environmental water samples. FQs in water samples were selectivity captured by the MIPs material, held on a syringe filter and directly online eluted by the electrospray solution for mass spectrometric analysis. One sample analysis time was less than 3 min, and consumes a volume of organic solvent less than 300 μL. The results demonstrated that MIPs-iEESI-MS/MS has advantages including easy use, high speed, high sensitivity for the analysis of FQs in environmental water, which has potential application in environmental science and water safety control.

## 2. Experimental

### 2.1 Reagents and apparatus

MIPs material (SupelMIP™ SPE-Fluoroquinolones) was purchased from Sigma-Aldrich (St. Louis, MO, USA) with average size of 61 μm. The syringe filter (aperture size of 0.22 μm) was purchased from Tianjin Navigator Lab Instrument Co., Ltd (Tianjin, China). Fluoroquinolones (fleroxacin, norfloxacin, and enoxacin, purity 98%) were purchased from J&K Scientific Ltd (Shanghai, China). The individual working stock solutions of fleroxacin, norfloxacin, and enoxacin, were prepared in methanol at a concentration of 0.1 mg mL^−1^ and stored at 4 °C before use. Working solutions were freshly prepared daily by diluting the stock solutions with deionized water. Methanol (purity 99.9%) was purchased from Merck KGaA (Darmstadt, Germany) and ammonium hydroxide solution (20–22%, w/w) was purchased from CNW Technologies GmbH (Düsseldorf, Germany). Deionized water was obtained from a Millipore water purification system (Milli-Q, Millipore; Bedford, MA, USA).

**Fig. 1:**
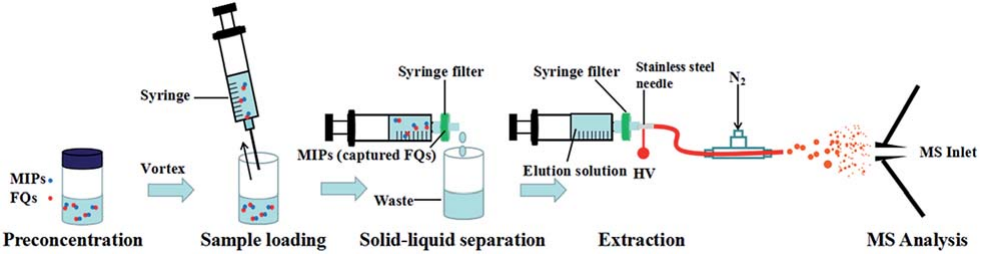
Schematic illustration of MIPs-iEESI-MS for analysis of FQs.

All the experiments were performed using an Orbitrap Fusion™ Tribrid™ mass spectrometer (Thermo Scientific, San Jose, CA, U.S.A.) coupled with a homemade MIPs-iEESI source. The homemade iEESI source was constructed for the online extraction and detection of FQs in water samples, and it was composed of a syringe filter, a stainless steel needle, a capillary and a gas path of N_2_. The connection sequence was shown in Fig. 1. Mass spectra were collected in the mass range of *m*/*z* 50–500 with positive ion detection mode. The ionization voltage was set at +3.0 kV and the heated ion capillary was maintained at 250 °C. The electrospray solution was pumped at a flow rate of 8 μL min^−1^ using a disposable syringe (Hongda Company, Nanchang, China) and the pressure of nitrogen sheath gas was 60 Arb. Collision induced dissociation (CID) experiments were carried out for MS/MS analysis. During the CID experiments, the precursor ions were isolated with a mass-to-charge window width of 1.0 Da, and then subjected to CID with collision energy of 30–40%. Other parameters were set to default instrument values.

### 2.2 Water samples

Environmental water samples were collected from four different rivers (Songhua River, Xilin River, Zhaosutai River and Dongliao River) in Jilin province, China. All the environmental samples were directly used for the MIPs-iEESI-MS/MS analysis after simple sedimentation in the bottle.

### 2.3 MIPs-iEESI-MS/MS analysis

A diagram describing the steps of the MIPs-iEESI-MS/MS produce was given in Fig. 1. Firstly, the 1 mL water sample was added into a 5 mL glass vial with 1 mg MIPs inside. Subsequently, the mixture was vigorously vortexed about 30 s using a Lab Dancer (IKA, Germany). Owing to the property of selectivity and specificity, the FQs molecules could be captured by the MIPs material. Then, the sample mixture was loaded in a 1 mL syringe (Hongda Company, Nanchang, China) and pumped out through an syringe filter, which made MIPs material capturing the FQs purposely stay on the syringe filter and achieved the solid–liquid separation. Finally, MIPs material staying on the syringe filter was washed by 1 mL deionized water to minimize the matrix interference. During the extraction process, 300 μL elution solution (2% ammonia in methanol, v/v) was employed to desorb the FQs from the MIPs material, forming a FQs contained eluent for electrospray purpose.

## 3. Results and discussion

### 3.1 Qualitative detection of FQs in water samples by MIPs-iEESI-MS/MS

**Fig. 2:**
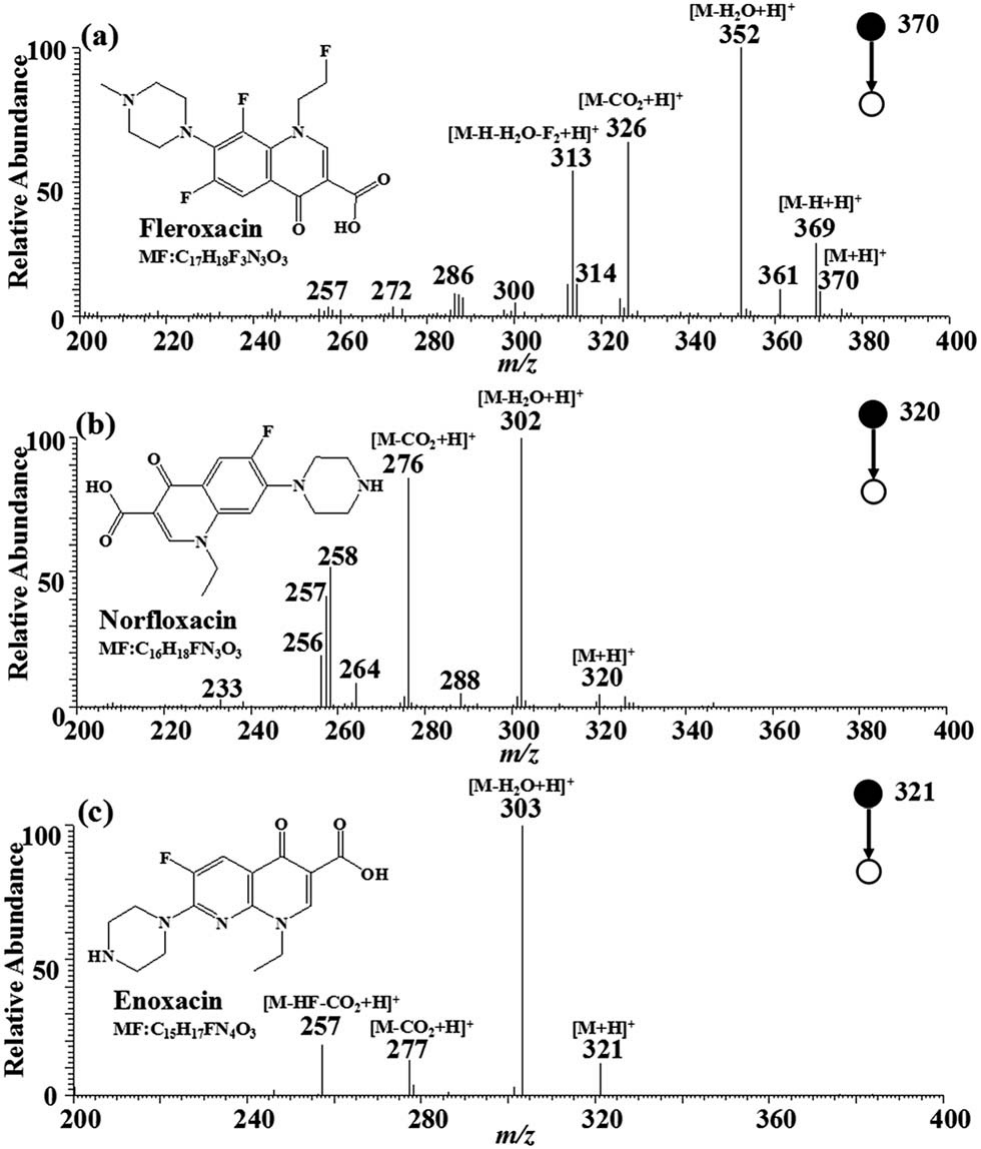
MIPs-iEESI-MS/MS spectra of FQs spiked in deionized water at the concentration of 10 μg L^−1^. (a) Fleroxacin, (b) norfloxacin and (c) enoxacin.

The MS^2^ strategy was utilized to further enhance the identification of the three target FQs. The MIPs-iEESI-MS/MS CID experiments were performed by using a spiked blank water sample at the FQs concentration of 10 μg L^−1^. Fleroxacin (MW 369.13) is an important antibiotic belonging to fluoroquinolones, and has been used in empirical treatment of a variety of infections, particularly infection of the genitourinary, gastrointestinal, and respiratory tracts.^35^ In the full-scan mass spectrum, the protonated molecular ion of fleroxacin at *m*/*z* 370 can be observed. In its MS^2^ mass spectrum (Fig. 2a), the protonated fleroxacin forms the ion at *m*/*z* 352 ([M–H_2_O + H]^+^) easily by lossing a water molecule, and by lossing a carbon dioxide molecule to generate the ion at *m*/*z* 326 ([M–CO_2_ + H]^+^). The fragment ion at *m*/*z* 313 ([M–H_2_O–H–F_2_ + H]^+^) is likely formed by lossing a water molecule, two fluorine atoms and a hydrogen atom from protonated fleroxacin. Norfloxacin (MW 319.13), the antibacterial synthetic drug, belongs to the third generation of FQs. The protonated amantadine molecular ion at *m*/*z* 320 can be observed in the full-scan mass spectrum. The main fragment ions of protonated amantadine, corresponding to peaks at *m*/*z* 306 ([M–H_2_O + H]^+^) and *m*/*z* 276 ([M–CO_2_ + H]^+^), can be found in the tandem mass spectrum (Fig. 2b). Enoxacin (MW 320.12) belongs to the third generation of multiple fluorinated antibacterial quinolone derivatives, widely used in the treatment of systemic infections.^36^ The MS^2^ spectrum for protonated enoxacin in the positive ion mode is shown in Fig. 2c, which shows [M–H_2_O + H]^+^ at *m*/*z* 303 as the base peak accompanied by [M–CO_2_ + H]^+^ at *m*/*z* 277 and [M–CO_2_– HF + H]^+^ at *m*/*z* 257. The loss of CO_2_ (−44) seemed to provide higher characteristic significance for the identification of FQs. Thus, fragment ion of *m*/*z* 326, *m*/*z* 276, and *m*/*z* 277 were selected for quantitative analysis of fleroxacin, norfloxacin, and enoxacin. As a result, the FQs in the water sample were successfully detected with the MIPs-iEESI-MS/MS method.

### 3.2 Optimization of analytical conditions of MIPs-iEESI-MS/MS

To obtain better performance of MIPs-iEESI-MS/MS, the influence of experimental parameters, such as sorbent amount, composition and volume of elution solution, flow rate of elution solution, and the mixture vertexing time were optimized. All experiments were performed in six times, and the concentration of fleroxacin in the spiked environmental water samples was 1 μg L^−1^.

**Fig. 3:**
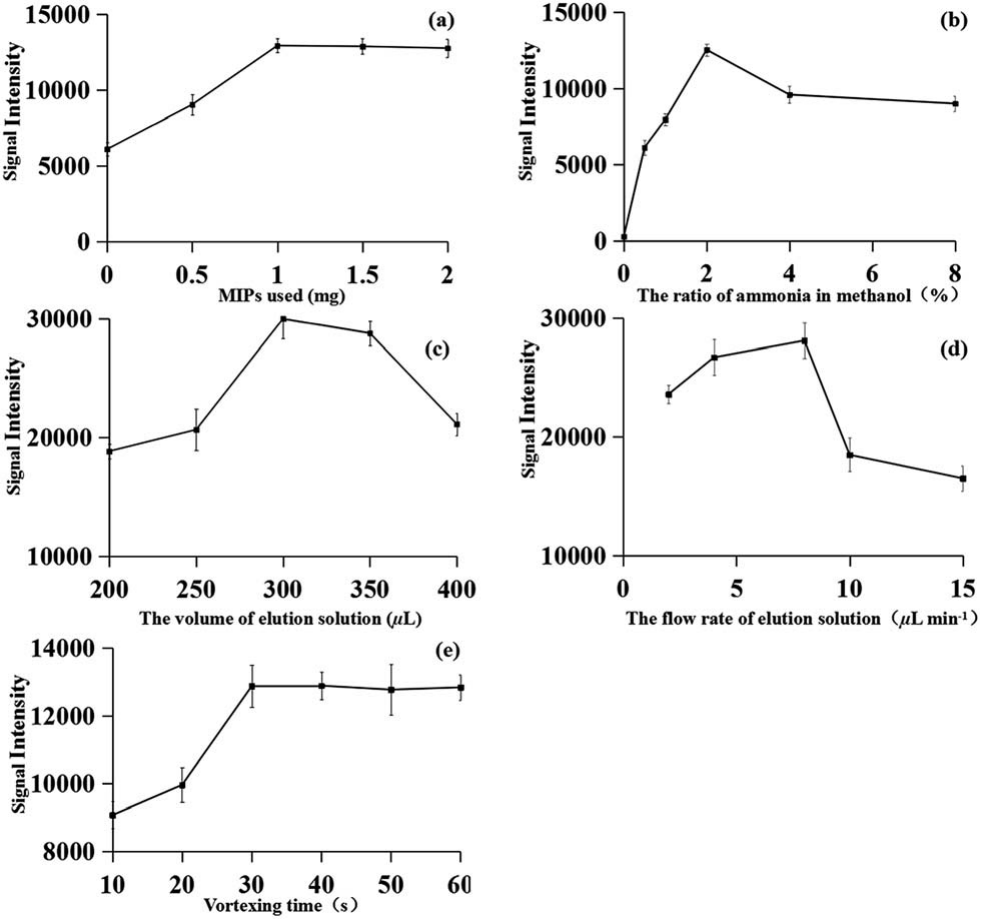
Optimization of the MIPs-iEESI-MS/MS experimental conditions: (a) amount of MIPs (b) composition of elution solution (c) volume of elution solution (d) flow rate of extraction and (e) mixture vortexes time. *n* = 6, the error bar represents a standard deviation of 6 determinations.

A comparison experiment of the MIPs material, C18, the graphene material and none adsorbent was carried out. All the experiments were carried out with the same samples by iEESI-MS/MS. As expected, the target FQs signals using MIPs material were remarkably higher than those using C18 and graphene (Table 1). To achieve high extraction efficiency for the fleroxacin, different amounts of MIPs material (*i.e.*, 0, 0.5, 1, 1.5, and 2.0 mg) were used for MIPs-iEESI-MS/MS analysis of fleroxacin. The signal intensity of fleroxacin increased with the increasing of MIPs amount from 0 to 1 mg and maintained at a high level signal intensity with MIPs amount over 1 mg, which showed a satisfactory performance with 1 mg MIPs material (Fig. 3a). Therefore, the optimized amount of MIPs (1.0 mg) was obtained.

**Table 1:** Comparison of the obtained signal intensity of the iEESI-MS/MS method when coupled with different kinds of adsorbents

Analytes	None adsorbent	C18	Graphene	MIPs
Fleroxacin^a^	103.47	1482.67	1951.33	3006.67
Norfloxacin^b^	19.91	174.70	180.33	358.67
Enoxacin^c^	15.88	127.83	149.50	181.17

a Blank water samples were spiked with 0.5 μg L^−1^ concentration of fleroxacin.

b Blank water samples were spiked with 0.5 μg L^−1^ concentration of norfloxacin.

c Blank water samples were spiked with 0.5 μg L^−1^ concentration of enoxacin.

The elution solution performed as both desorbing solutions for fleroxacin desorption from MIPs material and the solution for electrospray. Thus, the elution solution is of great significance for the improvement of MIPs-iEESI-MS/MS capacity. Methanol solutions containing with different proportion of ammonia 0%, 0.5%, 1.0%, 2.0%, 4.0%, and 8.0% (v/v) were employed as the elution solution for MIPs-iEESI-MS/MS. The signal intensity of fleroxacin was increased with the increasing of ammonia proportion from 0% to 2%, while the signal intensity presented a decreased trend when the ammonia proportion was excess of 2% (Fig. 3b). The ammonia should be helpful for desorption of fleroxacin from the MIPs material, while excessively high concentration of ammonia should suppress the ionization efficiency. Thus, 2% ammonia in methanol (v/v) was selected for all the MIPs-iEESI-MS/MS experiments.

The volume of the elution solution was optimized to improve the elution and ionization efficiency of MIPs-iEESI-MS/MS. An appropriate volume of elution solution is important to elute the trapped fleroxacin efficiently, but excessive volume elution solution will dilute the target analyte. So, higher signal intensity level was obtained with the volume of elution solution of 300 μL (Fig. 3c).

Also, in order to obtain a higher elution and ionization efficiency, the flow rate of elution solution (*i.e.*, 2, 4, 8, 10, and 15 μL min^−1^) was investigated. The signal intensity of fleroxacin was increased from 2 to 8 μL min^−1^ because of the increased elution rate. However, the signal intensity presented a decreased trend when the flow rate over 8 μL min^−1^ (Fig. 3d). So, the optimized flow rate of 8 μL min^−1^ was obtained.

Finally, the vortexing time (*i.e.*, 10, 20, 30, 40, 50, and 60 s) was investigated to obtain a satisfactory extraction and enrichment performance. As shown in Fig. 3e, higher signal intensity was appeared and kept a high level with the vortexing time over 30 s.

### 3.3 Matrix effects

Matrix effects from highly complex samples are a great challenge on the quantitative analysis of ambient mass spectrometry because of serious ion suppression or ion enhancement. To evaluate the matrix effects, calibration curves were compared and calculated between standard deionized water solutions and spiked environmental water sample (Songhua River) in a concentration range of 0.1–500.0 μg L^−1^ for fleroxacin, and 0.1–50 μg L^−1^ for norfloxacin and enoxacin.

Matrix effects were calculated by the following equation:
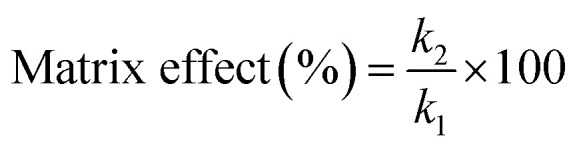
where *k*_2_ and *k*_1_ are the slope of calibration curves obtained by environmental water and deionized water.^37^ The matrix effects ranging from 94.83% to 102.26% for MIPs-iEESI-MS/MS as shown in Table 2. The results show that matrix effect of the MIPs-iEESI-MS/MS method exists but it is not serious. The values of *k*_2_/*k*_1_ close to 1, indicate that there are no significant differences between the two calibration curves.

**Table 2:** Analytical performance of MIPs-iEESI-MS/MS method

Analytes	Linear range (μg L^−1^)	Regression equation *y* = (*a* ± SD_*a*_)*x* + (*b* ± SD_*b*_)	Correlation coefficient	LOD (μg L^−1^)	Matrix effect (%)	*t* a
Fleroxacin	0.1–500.0	*y* = (1882.83 ± 7.64)*x* + (2173.66 ± 1581.37)	0.9999	0.015	99.33	1.891
Norfloxacin	0.1–50.0	*y* = (621.27 ± 6.66)*x* + (52.47 ± 149.65)	0.9995	0.026	102.26	2.210
Enoxacin	0.1–50.0	*y* = (262.70 ± 1.53)*x* + (67.52 ± 34.37)	0.9999	0.018	94.83	2.160

a *t*_0.01, 10_ = 2.764.

Moreover, to evidence the matrix effect, the regression parameters of the two calibration curves obtained by standard deionized water solutions and spiked environmental water sample (Songhua River) were compared by using Student's *t*-test. As shown in Table 2. The statistical analysis indicates that there are no significant differences among regression parameters obtained by standard deionized water solutions and spiked environmental water sample (*p* < 0.01). Thus, the calibration curves obtained by standard deionized water solutions were used for the reliable quantification.

### 3.4 Quantitative analysis of FQs in water samples by MIPs-iEESI-MS/MS

A series of water samples containing 0.1–500.0 μg L^−1^ of fleroxacin standard solutions were prepared as working solutions for the quantitative analysis of fleroxacin. The satisfactory result was obtained for the fleroxacin in the water samples and concentration range of 0.1–500.0 μg L^−1^ (0.1, 1, 5, 10, 100, 200 and 500 μg L^−1^), which showed a linear regression equation of *y* = (1882.83 ± 7.64)*x* + (2173.66 ± 1581.37) with a *R*^2^ value of 0.9999. The limit of detection (LOD) was determined at signal/noise ratio (S/N) = 3 and the recovery was calculated as the percentage of the measured spike of the matrix sample relative to the amount of spike added to the sample. A LOD of 0.015 μg L^−1^ was obtained for fleroxacin (Table 2). The recoveries were calculated in the range of 91.14–95.28% with the spiked concentrations of fleroxacin 0.5 μg L^−1^, 50 μg L^−1^ and 300 μg L^−1^ (Table 4). Then, series of water samples containing 0.1–50.0 μg L^−1^ of norfloxacin and enoxacin standard solutions were prepared as working solutions for the quantitative analysis. In the case of norfloxacin, the satisfactory result was obtained in the concentration range of 0.1–50.0 μg L^−1^ (0.1, 1, 5, 10, 20 and 50 μg L^−1^), which showed a linear regression equation of *y* = (621.27 ± 6.66)*x* + (52.47 ± 149.65) with a *R*^2^ value of 0.9995. A LOD of 0.026 μg L^−1^ was obtained (Table 2). The recoveries were calculated in the range of 103.51–103.60% with the spiked concentrations of norfloxacin 0.5 μg L^−1^ and 15 μg L^−1^ (Table 4). In the case of enoxacin, the satisfactory result was obtained in the concentration range of 0.1–50.0 μg L^−1^ (0.1, 1, 5, 10, 20 and 50 μg L^−1^), which showed a linear regression equation of *y* = (262.70 ± 1.53)*x* + (67.52 ± 34.37) with a *R*^2^ value of 0.9999. A LOD of 0.018 μg L^−1^ was obtained (Table 2). The recoveries were calculated in the range of 98.32–102.13% with the spiked concentrations of enoxacin 0.5 μg L^−1^ and 15 μg L^−1^ (Table 4). Four real environmental water samples were examined by the present method. No analyte ion peaks were observed in the mass spectra, indicating no residues of the three FQs at detectable levels in real samples. Furthermore, the FQs in the spiked water sample from Songhua River at different fortified concentrations were determined. It can be seen that the present method provides good recoveries (91.14–103.60%) and acceptable precision (<6.18%) (Table 4). These results indicated that the three FQs in water samples were effectively extracted and determined by the present method.

**Table 3:** Analytical performance of ESI-MS/MS method

Analytes	Linear range (μg L^−1^)	Regression equation *y* = (*a* ± SD_*a*_)*x* + (*b* ± SD_*b*_)	Correlation coefficient	LOD (μg L^−1^)	Matrix effect (%)
Fleroxacin	1.0–50.0	*y* = (8007.94 ± 368.59)*x* + (−4458.84 ± 9067.64)	0.9937	0.079	60.11
Norfloxacin	1.0–100.0	*y* = (1829.45 ± 99.37)*x* + (7145.69 ± 4559.52)	0.9912	0.233	68.79
Enoxacin	1.0–100.0	*y* = (1460.20 ± 82.64)*x* + (4566.55 ± 3791.74)	0.9905	0.111	70.41

**Table 4:** Recoveries obtained by MIPs-iEESI-MS/MS and ESI-MS/MS method

Analytes	MIPs-iEESI-MS/MS			ESI-MS/MS		
	Spiked concentrations (μg L^−1^)	Recovery (%)	RSD (%)	Spiked concentrations (μg L^−1^)	Recovery (%)	RSD (%)
Fleroxacin	0.5^a^	91.14	6.18	3	64.56	8.54
	50^a^	95.28	5.55	15	78.40	5.62
	300^a^	92.36	3.14	—	—	—
Norfloxacin	0.5^b^	103.51	3.75	3	15.66	5.93
	15^b^	103.60	5.74	15	91.94	3.00
Enoxacin	0.5^c^	102.13	4.30	3	16.17	7.48
	15^c^	98.32	4.24	15	73.43	7.34

a Blank water samples were spiked with a series concentration (μg L^−1^) of fleroxacin.

b Blank water samples were spiked with a series concentration (μg L^−1^) of norfloxacin.

c Blank water samples were spiked with a series concentration (μg L^−1^) of enoxacin.

### 3.5 Precision and accuracy

**Fig. 4:**
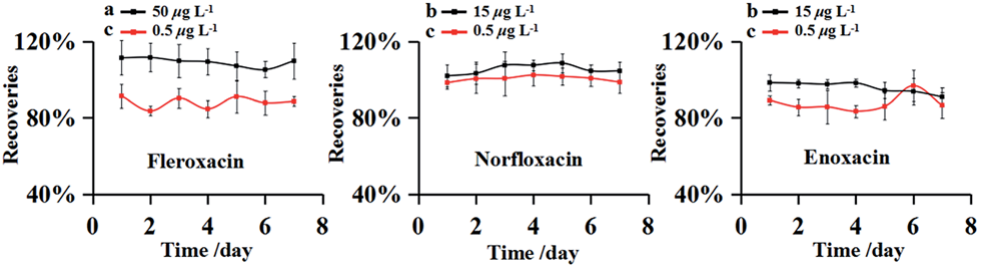
Recoveries curves of inter-day. Spiked sample at the concentrations of 50 μg L^−1^ (a), 15 μg L^−1^ (b) and 0.5 μg L^−1^ (c).

The precision of this method was evaluated by measuring the RSDs of the inter-day tests. The experiment were carried out with the FQs spiked at three different concentrations (0.5, 15 and 50 μg L^−1^) in environmental water sample from Songhua River. The spiked samples were analyzed in seven consecutive days and all experiments were performed in triplicate. The results obtained were shown in Fig. 4. Inter-day recoveries (accuracy) were obtained from 83.36 to 111.53% with the RSDs (precision) less than 8.83%. The recoveries kept at a steady trend in the inter-day recoveries curve, indicating satisfactory accuracy.

### 3.6 Comparison with ESI-MS/MS and other reported methods

For evaluating the MIPs-iEESI-MS/MS method performance, a comparison experiment of the proposed MIPs-iEESI-MS/MS method with a conventional ESI-MS/MS (direct injection without separation and enrichment) in the analysis of FQs was conducted. A series of water samples containing different concentrations of fleroxacin (1.0–50.0 μg L^−1^), norfloxacin and enoxacin (1.0–100.0 μg L^−1^) were prepared. MS^2^ fragment ions of *m*/*z* 326, *m*/*z* 276, and *m*/*z* 277 were selected for quantitative analysis of fleroxacin, norfloxacin, and enoxacin, respectively. The standard working curves for ESI-MS/MS method were constructed by plotting the signal intensity measured *versus* the concentrations of FQs in the spiked water samples. The slope and intercept of the linear regression equations and correlation coefficients are listed in Table 3. Good linearity was obtained in the range of 1.0–50.0 μg L^−1^ (1, 5, 10, 20 and 50 μg L^−1^) for fleroxacin and 1.0–100.0 μg L^−1^ (1, 5, 10, 20 and 100 μg L^−1^) for norfloxacin and enoxacin with correlation coefficients ranging from 0.9905 to 0.9937 for all the analytes. The LODs of the analytes were in the range of 0.079–0.233 μg L^−1^ for the three FQs. Furthermore, the FQs in the spiked environmental water samples at two different fortified concentrations of 3 and 15 μg L^−1^ were determined by using ESI-MS/MS method. The analysis process of environmental water samples was operated as follows: the water samples were centrifuged at 4500 × *g* at 5 °C for 10 min. Then 1.0 mL supernatant was passed through a 0.22 μm PTFE filter membrane and the resulting solution was used for ESI-MS/MS analysis. The results are listed in Tables 3 and 4, from which we can conclude that the MIPs-iEESI-MS/MS method obtained good recoveries and lower LODs, which also confirmed the sensitivity and propriety of the proposed method.

Furthermore, a comparison of the MIPs-iEESI-MS/MS method with other reported methods, including SPEs and MIP-SPE, for the determination of FQs and QAs in water was also made. The results are presented in Table 5. The data in Table 5 showed that the method established in this work obtained good sensitivity, and was of higher speed (less than 3 min per sample) than those previously reported methods. Therefore, MIPs-iEESI-MS/MS could be used as a simple, high-sensitivity and efficient method to detect FQs in environmental water samples.

**Table 5:** Comparison of proposed MIPs-iEESI-MS/MS method with other methods for the determination of FQs residues

Techniques	Samples	Analytes^a^	Time required	Determination	LODs	Ref
SPE	Water	FQs, SAs. *etc.*	>45 min	LC-ESI-MS/MS	0.6–8.1 μg mL^−1^	38
SPE	Water	QAs. etc	>25 min	LC-MS/MS	8.6–49 ng L^−1^	39
SPE	Water	FQs, QAs	>30 min	LC-MS/MS	0.6–50 ng L^−1^	40
MIP-SPE	Water, tissue	FQs	>1.5 h	LC-MS/MS	0.1–5 μg L^−1^	41
SPE	Water	FQs, SAs. *etc.*	>42 min	LC-MS/MS	0.01–3.73 μg L^−1^	42
MIPs-iEESI	Water	FLE, NOR. *etc.*	<3 min	iEESI-MS/MS	0.015–0.026 μg L^−1^	This work

a Abbreviations: FQs, fluoroquinolones; NOR, norfloxacin; FLE, fleroxacin; QAs, quinolone antibiotics; SAs, sulfonamides.

## 4. Conclusion

In this study, a rapid, high sensitivity, environmentally friendly and molecular specificity method based on MIPs material combined with iEESI-MS/MS was developed for the quantitative analysis of FQs in environmental water samples. Complex sample pretreatment processes such as filtration, purification or centrifugation was no need in the iEESI-MS/MS method, which greatly reduced the analysis cycle and improved the analysis efficiency. Moreover, the proposed method contains the advantages of low matrix effect, high speed and easy use. The developed MIPs-iEESI-MS for the rapid and accurate quantification of trace FQs in complex water samples may promote the application of fast mass spectrometry method in the environmental science and water safety control.

## Conflicts of interest

The authors declare that the research was conducted in the absence of any commercial or financial relationships that could be construed as a potential conflict of interest.

## Supplementary Material
